# Strengthening health systems through essential diagnostic lists and diagnostic network optimization

**DOI:** 10.1371/journal.pgph.0001773

**Published:** 2023-03-30

**Authors:** Kekeletso Kao, Mikashmi Kohli, Juhi Gautam, Hellen Kassa, Sam Acellam, Joseph Ndungu, Heidi Albert

**Affiliations:** 1 FIND, Geneva, Switzerland; 2 FIND India, Mumbai, India; 3 FIND Kenya, Nairobi, Kenya; 4 FIND South Africa, Cape Town, South Africa; Kamuzu University of Health Sciences (KUHeS), MALAWI

Diagnostics are an essential part of healthcare systems. We need diagnostics to confirm diseases, determine drug resistance, guide and monitor treatment, and for the surveillance of outbreaks. Universal health coverage cannot be achieved without ensuring that people have timely access to quality diagnostic services at all levels of the health system, without experiencing financial hardship [1]. However, despite government and multi-stakeholder efforts, diagnostics remain the weakest link in healthcare systems and the biggest gap in the care cascade for several diseases [2]. This is especially true in low- and middle-income countries (LMICs), where testing may be unavailable, unaffordable and underutilized [3]. Around half the world’s population has little to no access to diagnostics [2].

Many countries may have limited financial resources and infrastructure for diagnostics, and testing can be limited by siloed disease networks and inequitable geographic distribution of testing facilities. Effective diagnostic networks require an interconnected system of diagnostic testing devices, infrastructure and human resources to detect, manage and monitor diseases. As a result, it is important that countries have tools to guide the most effective use of resources, to ensure that people have access to high-quality, accurate, and affordable diagnostic testing when and where they need it.

**Fig 1:**
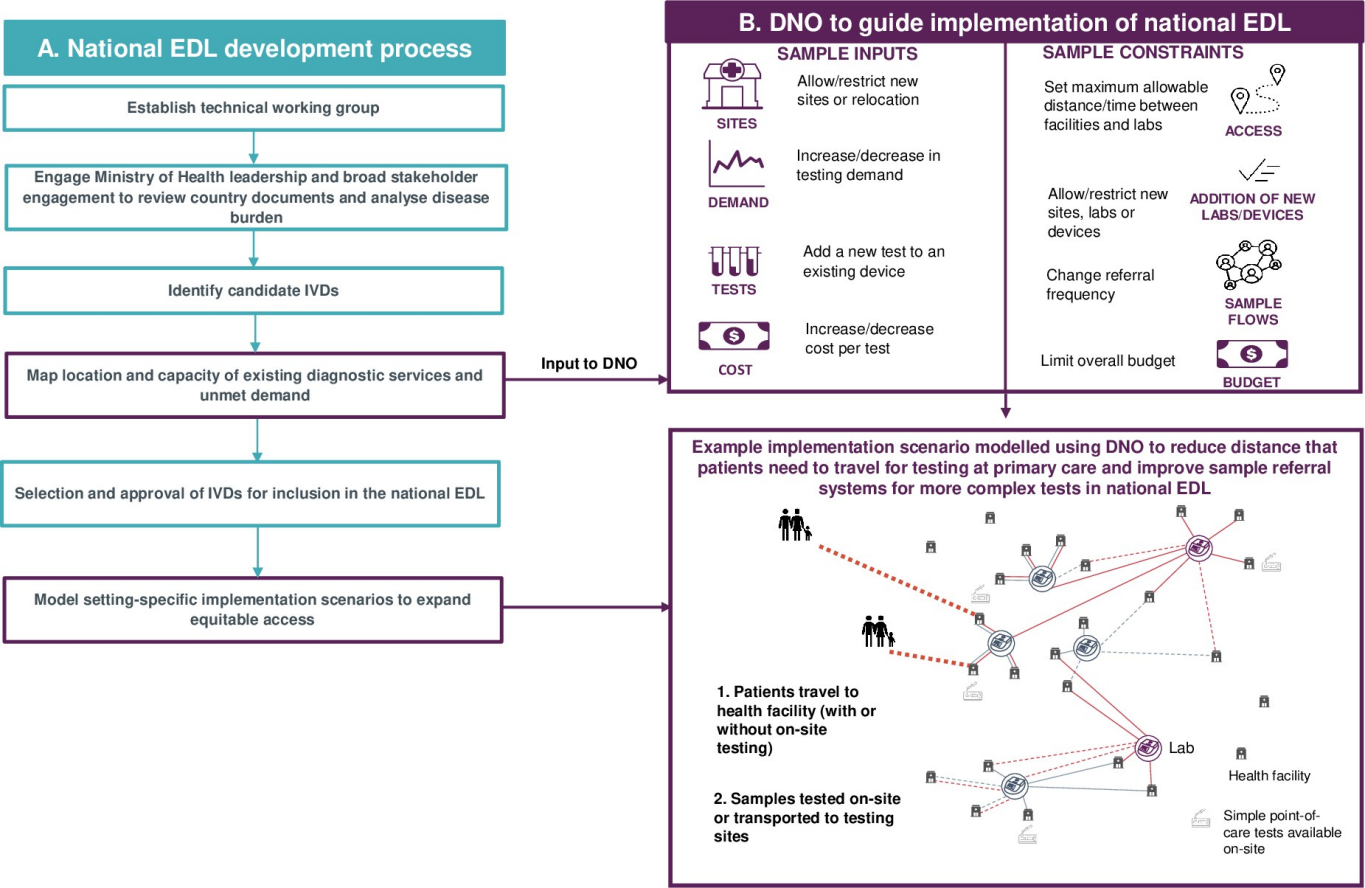
Overview of national essential diagnostic list development process and application of DNO. (A) Overview of national essential diagnostic list development process and example application of DNO to model optimized implementation of essential diagnostics (B). DNO, diagnostic network optimization; EDL, essential diagnostic list; IVD, in vitro diagnostic. In 2018, the World Health Organization (WHO) published their first model list of essential in vitro diagnostics, as an evidence-based list of essential in vitro diagnostics and recommended assay formats for those tests [5]. A third edition of the WHO EDL was published in 2021 [5]. This list serves as a reference and guidance document for programme and laboratory managers, procurement officers and other stakeholders to develop country-specific EDLs. The WHO EDL covers general diagnostic tests, disease-specific tests and tests for screening blood donations based on facilities with and without laboratories [5].

Two key tools that work in conjunction to support LMICs with strengthening access to diagnostics are essential diagnostic lists (EDLs) and diagnostic network optimization (DNO) [4–6]. An EDL lists categories of tests that should be available, as a priority, at different levels of a country’s healthcare system in response to common conditions and diseases of global importance [7]. National EDLs allow countries to select and place priority diagnostics that are appropriate for their disease burden, health infrastructure and current diagnostic capacity [8]. In the same way that essential medicines lists are intended to help ensure suitable access to and rational use of essential medicines [9], EDLs can promote affordable pricing of tests and help countries ensure appropriate and equitable access to diagnostics. The other key tool, DNO, is a geospatial analytics approach which uses geographic and health data to analyse the current diagnostic network in a country or region and develop networks that enable optimal access to testing for patients, taking into account disease burden, health system capacity and infrastructure [Fig 1]. The innovative DNO approach can be used to guide implementation of EDLs and ensure that a country’s diagnostic resources are implemented in the most effective manner.

Countries are expected to develop national EDLs that are tailored to their disease burden and health infrastructure, in the same way that over 150 countries have developed their own national lists of essential medicines from the WHO Model list of Essential Medicines [10]. Most countries will already have lists indicating diagnostics that should be made available at different tiers of the diagnostic network, e.g. diagnostics in essential health packages, diagnostic harmonization lists, and procurement lists, which may be used as a starting point for the development of national EDLs [7]. To date, India and Nigeria have developed national EDLs to increase access to affordable and appropriate diagnostic testing at the country level [6,11]. Efforts are also underway other countries including in Kenya, Malawi, South Africa, Ethiopia, the Gambia, Zimbabwe, Viet Nam and Indonesia to develop national EDLs supported by FIND, the global alliance for diagnostics, in collaboration with partners and under the leadership of Ministries of Health.

National EDLs can also help countries allocate budgets for priority diagnostics at different levels of the healthcare system. To support countries with developing national EDLs, WHO has issued technical guidance for countries around methods for developing and updating these documents [8]. The WHO guidance has led to the development by FIND and ASLM of a practical guidance document enabling countries to operationalize the technical guidance [12].

Once countries have developed a national EDL, the next critical step is implementing the recommended diagnostics as part of diagnostic networks, considering resource constraints and the country context. The innovative geospatial analytics approach of DNO provides an evidence-based methodology for countries to evaluate and design optimized diagnostic networks for a given setting. DNO provides possible solutions that can maximize patient access to services with cost efficiency and feasibility of implementation [3,13,14]. The approach has been successfully applied to optimize diagnostic networks for tuberculosis, antimicrobial resistance surveillance and neglected tropical diseases across a range of LMICs [15–17]. For example, in the Philippines, DNO was used to scale up and improve access to molecular testing for TB. The DNO analysis informed the procurement and placement of GeneXpert devices and the design of the associated sample referral system [13]. The analysis also informed the future integration of testing for HIV, human papillomavirus and SARS-CoV-2 [13].

DNO can support countries with implementing EDLs by enabling assessment of existing testing capacity and recommending the optimal diagnostic network configuration to deliver the required package of essential diagnostics, including for the most vulnerable populations, in a sustainable manner. The outputs can be used by decision-makers at the national, regional or district level in designing interventions to strengthen systems. DNO is of particular value when considering the integration of testing for different diseases, like COVID-19 and tuberculosis, on multi-disease platforms such as GeneXpert, and when considering integrating sample referral systems for different diseases. For example, in Zambia, DNO was applied to integrate HIV testing for pregnant and breastfeeding women and children with existing tuberculosis testing on GeneXpert machines [18]. Implementation of the integrated approach recommended by DNO was found to substantially improve access and same-day diagnosis of HIV for these priority populations and benefit the tuberculosis programmes [18]. When it comes to implementing national EDLs, DNO can be applied to model networks across healthcare tiers, from basic point-of-care tests at primary care through to tests requiring more complex and higher throughput instrumentation at higher tiers of the diagnostic network.

An open-access web-based tool, OptiDx, is available to support DNO analysis in LMICs [19]. OptiDx allows country-specific data around the diagnostic network to be entered and enables users to investigate the impact of changing different parameters of network design (e.g. the number of diagnostic devices, sample referral distance constraints) on the diagnostic network. FIND has also developed a guide to support countries with using DNO in their settings [13]. Other geospatial analytics tools, such as AccessMod, LabMap/PlanWise, can also provide valuable, complementary insights into the accessibility of diagnostic services, which together with DNO can provide a more holistic overview of the diagnostic network [13].

Gaps in the availability and accessibility of diagnostics are recognized as a barrier to achieving universal health coverage and the health-related Sustainable Development Goals [1,2]. Recovering from the impact of COVID-19, many LMICs face even greater pressure and have limited resources to strengthen diagnostic networks, although there may be a surplus of molecular diagnostics platforms that can be repurposed using DNO. Combining the power of EDLs to determine priority testing needs with the analytical approach of DNO can support countries in designing tailored diagnostic systems, thereby enabling optimal availability and access to diagnostics, in the most cost-efficient manner. In the future, it will be useful for countries that implement national EDLs and DNO to monitor the impact of the tools on diagnostic network performance and share lessons learned, to provide further evidence to guide wider adoption of the tools.
